# Resiliency Engagement and Care in Health (REaCH): a telephone befriending intervention for upskilled rural youth in the context of COVID-19 pandemic—study protocol for a multi-centre cluster randomised controlled trial

**DOI:** 10.1186/s13063-021-05465-5

**Published:** 2021-07-28

**Authors:** Saju Madavanakadu Devassy, Komal Preet Allagh, Anuja Maria Benny, Lorane Scaria, Natania Cheguvera, I. P. Sunirose

**Affiliations:** 1 Rajagiri College of Social Sciences (Autonomous), Rajagiri P.O, Kalamassery, Cochin, Kerala 683 104 India; 2 Rajagiri International Centre for Consortium Research in Social Care (ICRS), Cochin, Kerala India

**Keywords:** Randomised control trial, COVID-19, Befriending, India

## Abstract

**Background:**

The lockdown associated with the COVID-19 pandemic is likely to impact people’s mental health, especially those from economically disadvantaged and vulnerable sections of society. Mental health can be affected by many factors, including fear of disease transmission, from response measures against the pandemic like social distancing, movement restriction, fear of being in quarantine, loneliness, depression due to isolation, fear of losing work and livelihood and avoiding health care due to fear of being infected. Telephonic befriending intervention by non-specialists will be used to provide social and emotional support to the youth from the Deen Dayal Upadhyaya Grameen Kaushalya Yojana (DDUGKY), an initiative of the Government of India. This study aims to promote mental wellbeing and reduce depressive symptoms by assisting participants to mobilise social support from family, friends and significant others by using the telephonic befriending intervention.

**Methods:**

In this article, we report the design and protocol of a multi-centre cluster randomised controlled trial. In total, 1440 participants aged 18–35 years who have recently completed their course out of the DDU-GKY initiative will be recruited in the study from 12 project-implementing agencies (PIAs) across six geographical zones of India. Participants from 6 of these agencies will be assigned to the telephonic befriending intervention arm, and the other six agency participants will be assigned to the general enquiry phone call arm (control). The primary outcomes of this study are mental wellbeing, depressive symptoms and perceived social support. Baseline assessments and follow-up assessments will be carried out 1 month following the intervention using WHO-5, PHQ and MSPSS-12 questionnaires. The befriending intervention will be provided by DDU-GKY staff, whom a virtual training programme will train.

**Discussion:**

This trial will help assess whether participants who are offered emotional, social and practical support through befriending will experience lesser symptoms of depression and better mental health compared to participants who do not receive this intervention through mobilised social support from friends, family and others.

**Trial registration:**

Clinical Trial Registry India (ICMR-NIMS) CTRICTRI/2020/07/026834. Registered on 27 July 2020.

## Background

In India, the first case of COVID-19 was confirmed from the southern state of Kerala on 30 January 2020 [1]. On the 11th of March 2020, coronavirus disease (COVID-19) was declared a pandemic by the World Health Organization (WHO) [2]. Kerala declared a state-wide lockdown on the 23 March 2020 to prevent the community transmission of this virus [3]. As a result of the lockdown, people were forced to stay in their homes and faced with new realities of working from home, home schooling of children, temporary unemployment and lack of contact with other family members, friends and colleagues, which is likely to impact their mental and social health [4–7]. The unpredictability and uncertainty of controlling the disease and the misinformation around it will increase mental stress and concern among the people [4]. WHO defines mental health as a state of wellbeing in which an individual realises his or her own abilities, can cope with the everyday stresses of life, can work productively and can contribute to his or her community [8]. Recent studies have shown that even with a short-term lockdown, people report symptoms of mental stress and mental disorders like anxiety, depression, insomnia and post-traumatic stress disorder [5, 6]. The COVID-19 pandemic is likely to result in mental health problems varying from mild, time-limited distress to chronic, progressive and severely disabling conditions [9]. Findings from a recent online survey conducted among residents of New Delhi revealed that 55.3% of participants have sleep disturbances during the lockdown, 63.4% report work and income being severely affected, 7% report anxiety and restlessness and 12.1% felt helpless and depressed [10]. Thus, the pandemic is not just a threat to a country’s physical health but will also affect mental, social and economic well-being.

A survey by the Indian society of Labour economics showed job loss as the most severe immediate impact of COVID-19, while lower economic growth and rise in inequality as the probable long-term effects [11]. Loss of employment and the falling economy are expected to have a massive impact on mental health, especially those living under precarious conditions [12]. During such time, people experience fear, uncertainty, disruption, stress and other challenges that can trigger common mental health disorders like depression, anxiety and self-harm or suicidal behaviour [13–15].

The Ministry of Rural Development (MoRD), Government of India (GOI), launched an initiative in 2014 called the Deen Dayal Upadhyaya Grameen Kaushalya Yojana (DDU-GKY) to add diversity to the incomes of rural low-income families and to cater to the career aspirations of rural youth [16]. This initiative is present in 28 states of India and focuses on youth from poor rural families (aged 15 to 35 years). Currently, 1575 projects are being implemented by over 771 PIAs across India [16].

The Rajagiri College of Social Sciences, in Cochin, Kerala, is one of the PIAs, with 1500 students completing the course from 2016 till date, under the DDU-GKY initiative. This paper presents the study protocol of the REaCH (Resiliency Engagement and Care in Social Health) intervention trial. The ReaCH intervention trial involves using friendly and supportive telephonic interactions as an intervention for extending psychosocial support and creating a platform for sharing concerns of the youth, passed out from the DDU-GKY initiative, especially in the context of the COVID-19 pandemic and the associated lockdown.

People with socioeconomic vulnerability are more prone to common mental disorders like depression and anxiety [17–19], mainly due to isolation from inadequate social support and the absence of holistic health services [20]. Individuals from economically disadvantaged and vulnerable sections of society have been shown to appreciate and benefit from emotional and social support [21, 22]. One way of providing this support is through befriending, which has proven effective in improving mental health and reducing depression [21]. Befriending is defined as an intervention aimed at providing the client with additional social support by developing an empathic, non-judgmental, non-directive, affirming and purposeful relationship [21]. It involves a relationship between two individuals with regular input over a pre-specified period initiated and supervised by a third party [23]. Since the relationship is open-ended, it can develop over time from an artificial pairing of two people previously unknown to each other into a relationship in which the individuals are more comfortable sharing personal information [24]. Befriending is a non-directive emotional and social support typically administered by non-health professionals, and befriending conversations should avoid health-related matters and focus on enjoyable topics of interest to both parties [25].

In an epidemic outbreak, psychosocial interventions are essential [26] and social support has been shown to have a direct relationship with positive mental health and acts as a buffer against various stressors affecting mental health [27]. People with strong social relationships enjoy a sense of security and belonging to a network of systems that acts as a protective factor against various mental health issues [28].

Befriending is most often delivered by lay personnel or non-specialist workers, and [29] it is found that their use in the care of people with common mental disorders was cost-effective and cost-saving. In our trial, the intervention will be provided by DDU-GKY staff, which will enhance the existing staff, reduce cost, improve sustainability as the staff will be empowered to identify similar mental health issues in students in the future and increase acceptability as the staff are always available. Before the main trial, we plan a pilot trial among the students of the PIA at Rajagiri University, Kerala. Following the pilot trial, Rajagiri University team will lead the trial across 28 states of India, with the support of the Ministry of Rural Development. This nationwide telephonic befriending intervention is set out to address immediate priorities and strategies for providing emotional and practical support to the vulnerable people, in the context of COVID-19-related lockdown. We hypothesise that participants, whom we offer emotional, social and practical support through befriending, will experience lesser symptoms of depression and have better mental health compared to participants who do not receive this intervention.

## Methods

### Aim

The overall aim of the ReaCH intervention trial is to test the effectiveness of the befriending intervention among young adults in reducing mental health issues like anxiety, depression or suicidal behaviour in the context of the COVID-19 pandemic and the related restrictions. The befriending intervention will be used to determine if it can promote mental wellbeing and reduce depressive symptoms by assisting participants to mobilise social support from family, friends and significant others.

The objectives of this trial are:

- To proactively engage with students to hear their concerns in a non-judgmental manner and empathise with them.
- To reassure students about job loss/difficulty in finding a job and get them back to standard patterns of life, as early as possible
- To create a database of students who have either lost their jobs or are searching for jobs and linking them to the job market.
- To facilitate active linkage with various formal and informal resources to reduce their distress

### Design

Cluster randomised controlled trial

### Outcomes

1. Mental wellbeing measured by World Health Organization-Five Well-being Index (WHO-5) [30, 31]
2. Depressive symptoms measured by the Patient Health Questionnaire (PHQ-9) [32]
3. Perceived social support measured by the Multidimensional Scale of Perceived Social Support (MSPSS-12) [33]

### Study location

The entire country of India, comprising 29 states, is further divided into six geographical zones and decided to include PIAs representing each zone, in consultation with the Director of National Institute for Rural Development and Panchayat Raj (NIRDPR), training head and her team. This clustering was done to capture the sociocultural and economic variations in settings and population and to address the needs specific to each zone. Table 1 lists the states that fall under each zone. 771 DDUGKY project-implementing agencies are functioning in India, spread across the different states in each of the zones. The number of PIAs within each state varies, 8 being the lowest and 160 being the highest. A list of PIAs in each zone will be prepared, and 12 PIAs will be randomly selected from each zone.

**Table 1:** List of states that fall under each zone

Geographical zones	States in the zone
1. Northern zone	Delhi, Himachal Pradesh, Jammu and Kashmir, Punjab, Uttarakhand, Uttar Pradesh
2. North-East zone	Assam, Arunachal Pradesh, Manipur, Meghalaya, Mizoram, Nagaland, Sikkim, Tripura
3. Central zone	Madhya Pradesh, Chattisgarh
4. Eastern zone	Bihar, Jharkhand, Odisha, West Bengal
5. Western zone	Goa, Gujarat, Maharashtra
6. Southern zone	Andhra Pradesh, Karnataka, Kerala, Puducherry, Tamil Nadu, Telangana

### Recruitment of clusters (PIAs)

Two project-implementing agencies (PIA) will be recruited from each of the six zones (12 PIAs in total). For a PIA to be included in this study, it should be in one of the six zones and be an active, currently operational PIA with a full-time staff. A list of eligible PIAs will be prepared, and NIRDPR will use a computer-generated randomisation procedure to select the PIAs in the six zones. The NIRDPR will contact only those agencies meeting the inclusion criteria and invite them to participate in the study. This process will be continued till 12 consenting PIAs are selected for the project implementation. Six of the selected PIAs will be allocated to the intervention arm, and 6 PIAs will be allocated to the control arm. The state monitoring agencies will collect information on the number of full-time staff and contact details of students who completed their courses.

### Duration

The total duration of the trial is 4 weeks. The trial will run parallelly across all six zones. The expected duration of involvement of each participant is 1 month.

### Recruitment of participants within PIAs

We will recruit 120 passed out youth from each participating PIA (total= 1440). All participants from one PIA will be either in the intervention arm or control arm. The participants are aged between 18 and 35, of all genders, and upskilled in different domains. Participants will be invited to take part in baseline assessment and intervention programmes.

#### Inclusion criteria

1. Alumni students of the DDU-GKY programme who are either working or are in search of a job
2. Own a smartphone

#### Exclusion criteria

1. Do not possess a smartphone
2. Unable to operate a smartphone
3. Receipt of treatment for pre-existing mental health conditions in the last year

### Randomisation and blinding

Following the completion of the baseline assessment for all participants, the eligible participants will be randomised at a 1:1 ratio to one of the two arms (intervention or control) by the trial manager based on the PIA to which they belong. A computer technician will create a computer-generated random number list, and odd numbers on the list will be allocated to the intervention arm, and the even numbers will be allocated to the control arm.

### Design measures to avoid bias

The allocation codes will be concealed by the use of a computer-generated random number list. The principal investigator, the trial team, trial manager or staff members will be blind to the allocation codes during the trial. The control arm will also receive general enquiry phone calls that will further help in masking the allocation. To avoid contamination, we will physically separate the intervention team and the control team and blind the telephone support person (staff) about which arm they belong to. We believe the lockdown and work from home will assist in masking. Separate staff will make the phone calls in the intervention and control arms.

### Assessments and procedures

Baseline assessments will be administered to all participants through an online survey by the research team. Questionnaires include PHQ-9, MSPSS, WHO-5 and socio-demographics. These questionnaires will be translated into the local languages and back-translated for accuracy. Detailed instructions and sufficient explanations will be provided on the initial page of the online survey.

The PHQ-9 questionnaire will be used to measure depression, with scores of 1–4, 5–9, 10–14, 15–19 and 20–27 indicating minimal, mild, moderate, moderately severe and severe depressive symptoms. WHO-5 questionnaire will measure mental wellbeing and the total row score (ranging from 0 to 25) is multiplied by 4 to provide the final score. 0 represents the worst possible mental wellbeing, and 100 represents the best possible mental wellbeing. The MSPSS will be used to measure perceived social support from three sources: family, friends and a significant other. This measure contains 12 questions which rated on a 7-point scale as ‘very strongly disagree’, ‘strongly disagree’, ‘mildly disagree’, ‘neutral’, ‘mildly agree’, ‘strongly agree’ and ‘very strongly agree’.

Post-intervention follow-up assessment will be carried out through telephone for both control and intervention arm. This will use the same baseline survey assessment tools and will be performed online. The outcome assessors are professionals trained in research methodology and are specifically skilled in data collection and management.

### Training of the staff

The expert resource persons will train the chief executive officer and the chief operating officer/state programme manager from the six selected PIAs (12 in total) on the intervention manual’s content, process and strategies. Training of trainers will be given to 36 facilitators, six members each from 6 intervention PIAs and the State Rural Livelihood Mission (SRLM) from the selected states. A virtual master class will train these facilitators on providing the intervention (telephonic befriending). This training will last for approximately 6 h and will include a session on question and answers. Following this training, the training video, protocol booklet and frequently asked Q&As will be shared with the facilitators. Additionally, an online support centre will be set up for continuous support to the trainers.

The trained facilitators will train 6 PIA staff (two from each agency) and each trained PIA staff will train an average of 6 other staff members. Staff who are a part of the DDU-GKY project for 1 year or longer will be trained to deliver the intervention. Staff who are a part of the intervention team will only receive this training on the intervention module. These trained staff members will reach out to 720 DDU-GKY alumni through this intervention.

Each befriender will be allocated between 10 and 15 students in their PIA. A 6-h virtual training is designed for the staff, covering the components to be delivered during each session. An intervention manual for the training has been developed, which was also translated into the local language. After the training, the trained staff will be provided with the intervention manual, an online video of the training session with subtitles, audio clips of sample interview content and a module on frequently asked questions. The intervention manual includes guidelines on developing a relationship with the client, introduction and orientation of participant to befriending, management of participant distress, confidentiality and safety issues for both staff and participants. In addition to the virtual training, befrienders will be monitored frequently through the online support centre to ensure fidelity to the intervention protocol.

Separate staff members will be involved in the intervention arm and control arm. No specific training will be given for the staff making the telephonic calls in the control arm. They will provide general information about the precautions necessary to protect themselves from the infection and inquire about how the family is coping with the lockdown related issues.

### Intervention

The two arms of the trial are:

1. Telephone befriending intervention arm (*n*= 720)
2. General enquiry phone call arm (control) (*n*=720)

#### Intervention arm: telephone befriending intervention

**Fig. 1:**
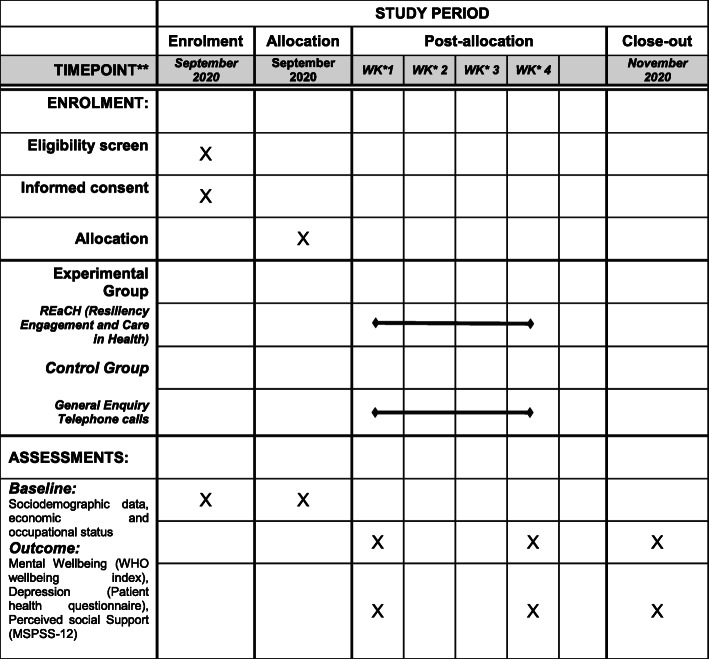
Schedule of enrolment, interventions and assessment (formatted on the basis of the Standard Protocol Items Recommendations for Interventional Trials [SPIRIT] 2013 template). *WK, week

**Fig. 2:**
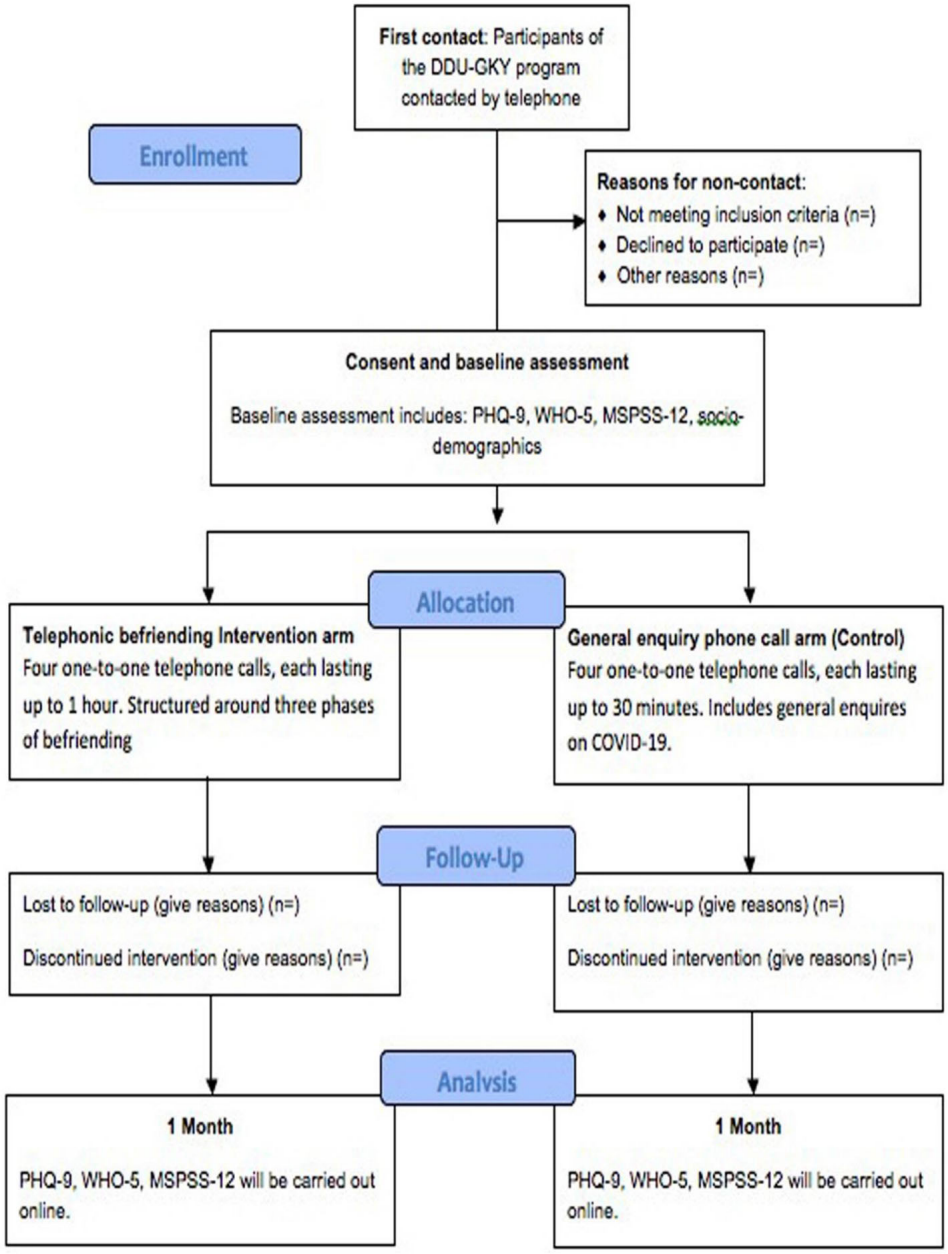
Process of the trial

The process of enrollment, allocation, follow-up and analysis is demonstrated (Figs. 1 and 2). The intervention involves participants receiving befriending from trained DDU-GKY staff, through one-to-one phone calls which they will receive in their homes, at a convenient time. The telephonic befriending intervention will be conducted in three phases: (i) proactive engagement and crisis intervention, (ii) problem-solving oriented support therapy and (iii) assertive linkage with community resources (see Table 2). The three phases will spread across four phone calls for 30 min to 1-h duration (Figs. 1 and 2). Their telephone support person will contact participants, and an introductory session will be arranged over the phone at each participants’ convenient time to establish a rapport.

**Table 2:** Three phases of the structured befriending intervention

Phase	Objectives	Interventions
**Phase 1**: Proactive engagement and crisis intervention	— To provide emotional support — To provide strategies to alleviate distress and crisis	In this session the befriender will: — Establish a positive and supportive relationship with the participant, — Provide emotional support — Psycho-education about COVID-19 including precautionary measures, — Enquire about general health and biological functioning.
**Phase 2**: Problem-solving oriented supportive therapy	— To provide strategies to solve client’s problems	This includes five general stages: — Problem orientation: Helping participant to identify the most distressing problem — Problem definition and formulation: defining the problem based on its impact on day to day functioning — Generation of alternatives: Helping the participant to identify alternative options — Assist the decision-making — Propose strategies to implement
**Phase 3**: Assertive linkage with community resources	— To utilise community resources for the well-being — To solve a crisis like job loss, financial insecurity, etc.	In this session, the befriender will: — Provide practical support and information; — Emphasise on social support and will provide strategies to improve it, — Reassurance to the participant before termination of the session.

During the telephone calls, a semi-structured questionnaire will be used to gather information on the stressors and risk factors in the context of this pandemic outbreak. Telephonic conversations will be recorded with the consent of the participants and later transcribed by the research team. The specific domains that will be assessed include the situational stressors in the context of the COVID-19 outbreak and the different support mechanisms available to manage the difficulties. The staff will be trained to encourage the participant to speak about their issues and difficulties in the present context and empathetically listen to their worries.

#### Control arm: general enquiry phone calls

Participants randomised to the control arm will not be receiving any intervention. However, they will participate in a baseline and follow-up assessment using the same instruments. They will receive four general enquiry phone calls lasting 5 to 30 min. It will be a general inquiry about the precautions that are necessary to protect themselves from the infection, and how the family is coping with the lockdown-related issues. The main focus will be given on psycho-education-based inquiries on COVID-19.

### Data entry and data management

The online data collection tool was developed and linked to the dedicated server in the research centre. The participant will enter the data directly to the online mobile portal, uploading the data directly onto the research server. The stored data set and codes will be protected using a password by the trial manager, independent of the trial team. The centrally stored data will be cleaned and analysed by the research team under the supervision of the trial manager, and the trial manager will verify the results independently to ensure the accuracy of the results. Data will be de-identified to ensure the confidentiality of information. Significant findings of the study will be reported in scientific journals and will be presented in conferences and workshops. Interested researchers can contact the principal investigator for further research collaborations.

### Sample size and statistical analysis

A target sample size of 1440 participants (720 in each arm) with less than 5% loss to follow-up after 1 month of intervention is estimated to have 80% power. All participants will be included in the analysis according to their allocated group at randomisation. Statistical tests will use a *p*-value less than 0.05 for significance. All statistical analysis procedures will be done using STATA 14 and R version 3.6.3. Baseline summary statistics (mean, standard deviation, percentage) will be calculated based on groups. Chi-square tests and *T*-tests will be performed to test the significance of the study variables. The odds ratio of the outcome variables for the post-assessment will be calculated using logistic regression modelling and 95% confidence intervals. We will analyse descriptive summaries of socio-demographic aspects and the scores of WHO-5, PHQ-9 and MSPSS as the baseline and after 1 month.

## Discussion

To the best of our knowledge, no previous published study or trial employs telephonic befriending for managing the psychological and social determinants of vulnerable groups with a country-level focus. Before the main trial, we have planned a pilot trial that will provide preliminary evidence regarding the feasibility, acceptability, and efficacy of a telephonic structured befriending intervention. It will explain to what extent a befriending intervention aids to reduce worry, unprecedented pressure and promote healthy coping to the challenges faced during the pandemic. The analysis from the main trial will aim to establish whether there are benefits from a telephone befriending intervention compared with the control group during a pandemic. During the COVID-19 pandemic, although maintaining physical distance is a necessity, being socially connected and closely in dialogue has become the need for the day.DDUGKY has a service support reputation where the PIAs have a post-placement tracking system for providing retention support to its students. This support is linked to financial incentives, accreditations and ratings to the PIAs, motivating them to deliver it effectively. This continued relationship will ensure a low loss to follow-up rate in this trial.

## Trial status

This intervention trial is registered under Clinical Trial Registry India (ICMR-NIMS) on 27 July 2020, with the registration number (CTRICTRI/2020/07/026834) and reference number (REF/2020/07/035397). The start date is 7 August 2020, and the whole process is likely to be completed by 31 July 2021. Recruitment will start from 1 September 2020 and will be completed by 10 September 2020.

## Data Availability

Not applicable

## Abbreviations

COVID-19: Coronavirus disease 2019
WHO-5: World Health Organization-Five Well-being Index
PHQ-9: Patient Health Questionnaire
MSPSS-12: Multidimensional Scale of Perceived Social Support
DDUGKY: Deen Dayal Upadhyaya Grameen Kaushalya Yojana
NIRDPR: National Institute for Rural Development and Panchayath Raj
PIA: Project Implementing Agency Declarations
